# De-identifying free text of Japanese electronic health records

**DOI:** 10.1186/s13326-020-00227-9

**Published:** 2020-09-21

**Authors:** Kohei Kajiyama, Hiromasa Horiguchi, Takashi Okumura, Mizuki Morita, Yoshinobu Kano

**Affiliations:** 1 Faculty of Informatics, Shizuoka University, Johoku 3-5-1, Naka-ku, Hamamatsu, Shizuoka, 432-8011 Japan; 2 National Hospital Organization Headquaters, 2-5-21 Higashigaoka, Meguro-ku, Tokyo, 152-8621 Japan; 3 National University Corporation Kitami Institute of Technology, 165, Koencho, Kitami, Hokkaido 090-8507 Japan; 4 Graduate School of Interdisciplinary Science and Engineering in Health Systems, Okayama University, 2-5-1, Kita-ku, Okayama, Okayama 700-8558 Japan

**Keywords:** De-identification, Electronic health records, Japanese language

## Abstract

**Background:**

Recently, more electronic data sources are becoming available in the healthcare domain. Electronic health records (EHRs), with their vast amounts of potentially available data, can greatly improve healthcare. Although EHR de-identification is necessary to protect personal information, automatic de-identification of Japanese language EHRs has not been studied sufficiently. This study was conducted to raise de-identification performance for Japanese EHRs through classic machine learning, deep learning, and rule-based methods, depending on the dataset.

**Results:**

Using three datasets, we implemented de-identification systems for Japanese EHRs and compared the de-identification performances found for rule-based, Conditional Random Fields (CRF), and Long-Short Term Memory (LSTM)-based methods. Gold standard tags for de-identification are annotated manually for *age, hospital*, *person*, *sex*, and *time*. We used different combinations of our datasets to train and evaluate our three methods. Our best F1-scores were 84.23, 68.19, and 81.67 points, respectively, for evaluations of the MedNLP dataset, a dummy EHR dataset that was virtually written by a medical doctor, and a Pathology Report dataset. Our LSTM-based method was the best performing, except for the MedNLP dataset. The rule-based method was best for the MedNLP dataset. The LSTM-based method achieved a good score of 83.07 points for this MedNLP dataset, which differs by 1.16 points from the best score obtained using the rule-based method. Results suggest that LSTM adapted well to different characteristics of our datasets. Our LSTM-based method performed better than our CRF-based method, yielding a 7.41 point F1-score, when applied to our Pathology Report dataset. This report is the first of study applying this LSTM-based method to any de-identification task of a Japanese EHR.

**Conclusions:**

Our LSTM-based machine learning method was able to extract named entities to be de-identified with better performance, in general, than that of our rule-based methods. However, machine learning methods are inadequate for processing expressions with low occurrence. Our future work will specifically examine the combination of LSTM and rule-based methods to achieve better performance.

Our currently achieved level of performance is sufficiently higher than that of publicly available Japanese de-identification tools. Therefore, our system will be applied to actual de-identification tasks in hospitals.

## Background

Recently, more electronic data sources are becoming available in the healthcare domain. Utilization of electronic health records (EHRs), with their vast amounts of potentially useful data, is an important task in the healthcare domain. New legislation in Japan has addressed the treatment of medical data. The “Act on the Protection of Personal Information [1]” was revised in 2017 to stipulate that developers de-identify “special care-required personal information.” This legislation further restricts the use of personal identification codes including individual numbers (e.g. health insurance card numbers, driver’s license card numbers, and governmental personnel numbers), biometric information (e.g. fingerprints, DNA, voice, and appearances), and information related to disability. This legislation can be compared with the “Health Insurance Portability and Accountability Act (HIPAA) [2]” of the United States, in that the Japanese Act in 2017 includes additional codes, with abstract specifications such as “you should strive not to discriminate or impose improper burdens,” and with exclusion of birth dates and criminal histories, as stipulated by HIPAA. Another related act of Japanese legislation, the “Act on Anonymously Processed Medical Information to Contribute to Medical Research and Development [3]” was established in 2018. This legislation allows specific third-party institutes to handle EHRs, thereby promoting wider utilization of medical data.

De-identification of structured data in EHRs is easier than that of unstructured data because it is straightforward to apply de-identification methods to structured data such as numerical tables. Although de-identification of unstructured data in EHRs is necessary, it is virtually impossible to de-identify the huge number of documents manually.

Several earlier works have examined EHR de-identification. The Informatics for Integrating Biology & the Bedside (i2b2) task [4] in 2006 was intended for automatic de-identification of clinical records to satisfy HIPAA requirements [2]. An earlier study prepared 889 EHRs, comprising 669 EHRs for training and 220 EHRs for testing. Their annotations included 929 *patient* tags, 3751 *doctor* tags, 263 *location* tags, 2400 *hospital* tags, 7098 *date* tags, 4809 *id* tags, 232 *phone_number* tags, and 16 *age* tags. The best performing method of i2b2 incorporated diverse features such as a lexicon, part-of-speech identification, word frequencies, and dictionaries for learning using an ID3 tree learning algorithm.

Grouin and Zweigenbaum [5] prepared 312 cardiovascular EHRs in French, with 3142 tags annotated by two annotators (kappa = 0.87). Their tags include 238 *date* tags, 205 *last_name* tags, 109 *first_name* tags, 43 *hospital* tags, 22 *town* tags, 8 *zip_code* tags, 8 *address* tags, 8 *phone* tags, 8 *med_device* tags, 3 *serial_number* tags. Of the *person* tags, 75% were replaced with other French person names. The other 25% were replaced with international names. They also collected 10 photopathology documents, for which a single annotator assigned 29 *date* tags, 68 *last_name* tags, 53 *first_name* tags, 17 *hospital* tags, 17 *town* tags, 13 *zip_code* tags, 14 *address* tags, 1 *phone* tag, 1 *med_device* tag, and 7 *serial_number* tags. They performed de-identification experiments using 250 documents as their training data and 62 documents as their test data for the cardiology corpus. They obtained better F1-scores (exact match, 0.883; overlap match, 0.887) using conditional random fields (CRF) than they obtained using their rule-based method (exact match, 0.843; overlap match, 0.847). However, their rule-based method was better for the photopathology corpus (exact match, 0.681; overlap match, 0.693) than their CRF-based method (exact match, 0.638; overlap match, 0.638) because the data were fewer than those of the cardiology corpus.

Grouin and Névéol [6] discussed annotation guidelines for French clinical records. After collecting 170,000 documents of 1000 patient records from five hospitals, they first prepared a rule-based system and their CRF-based system from their earlier study [5], which we described earlier. Their rule-based system relies on 80 patterns specifically designed to process the training corpus, and lists which they gathered from existing resources from the internet. They randomly selected 100 documents (Set 1) from their dataset and applied both systems. For each document, they randomly showed one output of the two systems to the annotators for revision. They applied their rule-based system to another set of 100 documents (Set 2), which were further reviewed and revised by a human annotator. They re-trained their CRF-based system using the revised Set 2 annotations, which is further applied to the other set of 100 documents (Set 3). Annotators reviewed these annotations in subsets for different agreement analyses. The study also compared human revision times among different annotation sets, which was a main objective of their study. They annotated 99 *address* tags, 101 *zip_code* tags, 462 *date* tags, 47 *e-mail* tags, 224 *hospital* tags, 59 *identifier* tags, 871 *last_name* tags, 750 *first_name* tags, 383 *telephone* tags, 218 *city* tags, in Set 1. They reported their rule-based method as better (0.813) in terms of the F1-score than their CRF-based method (0.519) when evaluated with 50 documents in Set 1. When trained with Set 2, the corpus of the same domain, their CRF-based system performed better, yielding 0.953 for Set 3 and 0.888 for Set 1 in their F1-scores.

From the Stockholm EPR [7], a Swedish database of more than one million patient records from two thousand clinics, Dalianis and Velupillai [8] extracted 100 patient records to create gold standard for automatic de-identifications based on HIPAA. They annotated 4423 tags, including 56 *age* tags, 710 *date_part* tags, 500 *full_date* tags, 923 *last_name* tags, 1021 *health_care_unit* tags, 148 *location* tags, and 136 *phone_number* tags. They pointed out that Swedish morphology is more complex than that of English. It includes more inflections, making the de-identification task in Swedish more difficult.

Jian et al. [9] compiled a dataset of 3000 documents in Chinese. It comprises 1500 hospitalization records, 1000 summaries, 250 consulting records, and 250 death records. They extracted 300 documents from this dataset randomly, discussed a mode of de-identification with lower annotation cost. They annotated their tags to these 300 documents (kappa = 0.76 between two annotators for their 100 document subset). Then they applied their pattern-matching module to these 300 documents, yielding a dense set of 201 sentences that include PHI (Protected Health Information). These 201 sentences included 141 name tags, 51 address tags, and 22 hospital tags.

Du et al. [10] conducted de-identification experiments using 14,719 discharge summaries in Chinese: two students annotated 25,403 tags. This dataset includes 6403 *institution* tags, 11,301 *date* tags, 33 *age* tags, 2078 *patient_name* tags, 3912 *doctor_name* tags, 326 *province* tags, 310 *city* tags, 774 *country* tags, 917 *street* tags, 277 *admission_num* tags, 21 *pathological_num* tags, 23 *x-ray_num* tags, 263 *phone* tags, 420 *doctor_num* tags, and 13 *ultrasonic_num* tags (inter-annotator agreement was 96%, kappa = 0.826). Their experiments demonstrated that their method of combining rules and CRF performed best, yielding a 98.78 F1-score. The Chinese language shares some issues with the Japanese language: they both require tokenization because no spaces exist between words. This issue makes de-identification tasks more difficult than they are in other languages.

The reports described above present a range of different evaluation scores. However they adopted different annotation criteria, which make direct comparison difficult. For instance, Grouin and Névéol used more detailed annotations than those used by Jian et al., as follows. Jian et al. introduced *Doctor* and *Patient* tags, but evaluated both simply as *Name*. Grouin and Névéol introduced *ZipCode, Identifier*, *Telephone*, and *City* tags, none of which is annotated in the work of Jian et al. Additionally, they assigned *Last Name* and *First Name* tags, where performance of *First Name* was better than *Last Name* by around 10 points. However, both are worse than the results reported by Jian et al., probably because Jian et al. applied their pattern-matching algorithm to filter their training data. Regarding *Address* tags, Jian et al. obtained a 94.2 point F-score, whereas the Grouin and Névéol CRF method obtained scores of fewer than 10 points. As Grouin and Névéol suggested, eliminating *City* tags in street names can greatly improve their results: their rule-based method yielded an 86 point F-score.

Unfortunately, automatic de-identification of EHRs has not been studied sufficiently for Japanese language. De-identification shared tasks for Japanese EHRs were held as tasks in MedNLP-1 [11]. Then named entity extraction was attempted in MedNLP-2 [12] tasks using datasets similar to MedNLP-1. We designate MedNLP-1 simply as MedNLP hereinafter because we specifically examine de-identification tasks but not other tasks held in the MedNLP shared task series.

Regarding machine learning methods, Support Vector Machine (SVM) [13] and CRF [14] were used often in earlier Named Entity Recognition (NER) tasks in addition to rule-based methods. Recent deep learning methods include Long-Short Term Memory (LSTM) [15] with character-embedding and word-embedding [16], which performed best for the CoNLL 2002 [17] (Spanish and Dutch) and CoNLL 2003 [18] (English and German) NER shared task data: these tasks require detection of “personal”, “location”, “organization”, and “other” tag types. Another LSTM model, which is similar to earlier work [16], was also applied to a task of NER from Japanese newspapers [19]. Although deep neural network models have been showing better results recently, rule-based methods are still often better than machine learning methods, especially when insufficient annotated data are available.

To evaluate the effectiveness of such different methods for the Japanese language, we implemented two EHR de-identification systems for the Japanese language in our earlier work [20]. We used the MedNLP shared task dataset and our own dummy EHR dataset, which was written as a virtual database by medical professionals who hold medical doctor certification. Based on this earlier work, we added a new dataset of pathology reports to this study, for which we annotated the following tags. De-identification tags of *age*, *hospital*, *sex*, *time*, and *person* are annotated manually in all these datasets, following the annotation standard of the MedNLP shared task to facilitate comparison with earlier studies. We assume these annotations as our gold standard for our de-identification task. To these three datasets, we applied a rule-based method, a CRF-based method, and an LSTM-based method. Additionally, we have annotated our own tags to these three datasets by three annotators to calculate inter-annotator agreement. We have observed the coherency of the original annotations of the datasets. Overall, this study differs from our earlier work [20] in that we added a new pathology dataset and its annotations, trained and evaluated our machine learning models using the new dataset, and evaluated the results using newly created annotations by three annotators to observe characteristics of the original and our own annotations.

## Datasets

Our datasets were derived from three sources: MedNLP, dummy EHRs, and pathology reports. Irrespective of the dataset source, de-identification tags of five types are annotated manually: *age* (numerical expressions of subject’s ages including its numerical classifiers), *hospital* (hospital names), *sex* (male or female), *time* (subject related time expressions with its numerical classifiers), and *person* (person names). Characteristics of these datasets are presented in Table 1. It is noteworthy that texts of the MedNLP and dummy EHRs are not actual texts, but they were written by medical professionals, each of whom holds medical doctor certification. However, characteristics of the descriptions differ between these two sources, probably because of differences of the writers. The number of annotators is not described for the MedNLP dataset, but a single annotator created the annotations of the dummy EHR dataset and the Pathology Report dataset, individually.

**Table 1:** Dataset characteristics

Dataset name	MedNLP	Dummy-EHRs	Pathology Reports
# of documents	50 reports	32 pairs of records and summaries	1000 reports
# of sentences	2244	8183	3012
# of tokens	42,621	154,132	194,449
# of all tags	490	3017	295
# of *age* tags	56	39	0
# of *hospital* tags	75	170	31
# of *person* tags	0	135	224
# of *sex* tags	4	16	0
# of *time* tags	355	2657	40
Example in original Japanese text	工場に勤めている<a > 64歳</a > の < x > 男性</x > 。	施設入所中で寝たきりの<a > 86歳</a > <x > 女性</x > 。全介助	<<院外標本 <h > 静大皮フ科クリニック</h > 、 < p > 桑田 智</p>
Example translated into English	A < a > 64-year-old</a > <x > man</x > works in a factory	An <a > 86-year-old</a > <x > woman</x > bedridden in a nursing home. Total assistance required	<<Ex-hospital sample < h > Shizudai Dermatology Clinic</h > , < p > Satoshi Kuwata</p>

### MedNLP shared task dataset

We used the MedNLP de-identification task dataset for comparison with earlier studies that have used the same dataset. This dataset includes the dummy EHRs (discharge summaries) of 50 patients. Although the training dataset and test dataset were provided from the shared task organizers, the test dataset of the formal run is not publicly available now. It is not possible to compare results directly with earlier works in the MedNLP shared task formal run (Tables 2 and 3 show the formal run results). However, both training and test datasets were originally parts of a single dataset. Therefore, we can discuss their characteristics in comparison with those found in earlier works conducted using the training dataset only. We calculated inter-annotator agreement by three annotators for the training dataset. The average F1-score of three pairs among these three annotators was 86.1, in 500 sentences of this dataset.

**Table 2:** Overall results

	*P*			*R*			*F*			*A*		
C3	89.59			91.67			90.62			99.58		
B3	91.67			86.57			89.05			99.54		
B1	90.05			87.96			88.99			99.49		
B2	90.82			87.04			88.89			99.52		
C1	92.42			84.72			88.41			99.49		
A1	91.50			84.72			87.98			99.47		
C2	91.50			84.72			87.98			99.46		
A2	90.15			84.72			87.35			99.41		
D1	86.10			74.54			79.90			99.36		
G1	82.09			76.39			79.14			99.38		
D3	85.87			73.15			79.00			99.35		
D2	80.81			74.07			77.29			99.24		
H2	76.17			75.46			75.81			99.28		
H1	75.81			75.46			75.64			99.27		
H3	74.88			74.54			74.71			99.26		

*P*, *R* and *F* were calculated at the phrase level: *P*, precision; *R*, recall; *F*, F1-measure; and *A*, accuracy. *A* was calculated in the word level (the agreement ratio of B-*, I-* and O).

The first column stands for participants’ team names, where the first letter stands for a team ID and the second numerical value stands for a submission run ID

**Table 3:** Detailed results for each privacy type in MedNLP-1 (*De-identification task*)

	<a > age			<x > sex			<t > time			<h > hospital name		
	*P*	*R*	*F*	*P*	*R*	*F*	*P*	*R*	*F*	*P*	*R*	*F*
C3	90.32	87.5	88.89	100	100	100	87.16	91.49	89.27	97.30	94.74	96.00
B3	90.00	84.38	87.10	100	50.00	66.67	91.30	89.36	90.32	97.06	86.84	91.67
B1	93.33	87.5	90.32	100	100	100	90.65	89.36	90.00	89.47	89.47	89.47
B2	90.00	84.38	87.10	100	100	100	91.24	88.65	89.93	91.89	89.47	90.67
C1	96.67	90.62	93.55	100	50.00	66.67	91.18	87.94	89.53	93.55	76.32	84.06
A1	92.86	81.25	86.67	100	50.00	66.67	91.04	86.52	88.73	91.89	89.47	90.67
C2	96.67	90.62	93.55	100	50.00	66.67	89.13	87.23	88.17	96.77	78.95	86.96
A2	92.86	81.25	86.67	100	50.00	66.67	89.05	86.52	87.77	91.89	89.47	90.67
D1	92.31	75.00	82.76	100	50.00	66.67	82.84	78.72	80.73	96.15	65.79	78.12
G1	80.65	78.12	79.37	100	50.00	66.67	84.56	81.56	83.03	72.73	63.16	67.61
D3	88.89	75.00	81.36	100	50.00	66.67	83.08	76.60	79.70	96.15	65.79	78.12
D2	92.31	75.00	82.76	100	50.00	66.67	75.86	78.01	76.92	96.15	65.79	78.12
H2	83.87	81.25	82.54	100	100	100	73.79	75.89	74.83	77.78	73.68	75.68
H1	80.65	78.12	79.37	100	100	100	75.86	78.01	76.92	70.27	68.42	69.33
H3	83.87	81.25	82.54	100	100	100	73.79	75.89	74.83	70.27	68.42	69.33

*P*, *R* and *F* were calculated at the phrase level: *P*, precision; *R*, recall; *F*, F1-measure; and *A*, accuracy. *A* was calculated in the word level (the agreement ratio of B-*, I-* and O).

The first column stands for participants’ team names, where the first letter stands for a team ID and the second numerical value stands for a submission run ID

### Dummy EHRs

Another source is our original dummy EHRs. We built our own dummy EHRs of 32 patients, assuming that the patients were hospitalized. Documents of our dummy EHRs were written by medical professionals (doctors). We added manual annotations for de-identification following the guidelines of the MedNLP shared task. These annotations were originally assigned by a single annotator. Additionally, we added new annotations by three annotators to a part of this dataset and calculated inter-annotator agreement. The average F1-score of three pairs among these three annotators was 76.1 for 730 sentences of the Dummy EHR dataset.

### Pathology reports

The other source is a dataset of 1000 short pathology reports, that differ greatly from the EHRs above. Pathology reports describe pathological findings by which personal information (names of patients, doctors, hospitals, and time expressions) frequently appears, but for which tags of *sex* and *age* rarely appear. Personal names, hospital names, and dates were manually de-identified beforehand by the dataset provider, and replaced with special characters. For machine learning methods to support realistic training and evaluation, we replaced these special characters with randomly assigned real entity names as follows. For the hospital names, we collected 96,167 hospital names which cover most of the Japanese hospital names, published by the Japanese government. For the person names, we manually created 20 dummy-family names and 20 dummy-first names using one of the last names only, or combining one of the last names and one of the first names. Additionally, we calculated the inter-annotator agreement by three annotators. The average F1-score of three pairs among these three annotators was 80.2 for 500 sentences of this dataset. This Pathology Report dataset is the only real (not dummy) dataset among our three datasets. Because we received manually de-identified version of the original real pathology reports, no ethical review was necessary.

## Methods

We used a Japanese morphological analyzer, Kuromoji,^1^ for tokenization and part-of-speech (POS) tagging. We registered our customized dictionary, derived from Wikipedia entry names and entries of the Japanese Standard Disease-code master [21], to this morphological analyzer in addition to the analyzer’s default dictionary.

We implemented rule-based, CRF-based, and LSTM-based methods.

### Rule-based method

Unfortunately, the implementation of the best system for the MedNLP-1 de-identification task [22] is not publicly available. We implemented our own rule-based program based on the descriptions in their paper, to replicate the same system to the greatest extent possible. We present their rules below for a target word x for each tag type.

#### Age

If x’s detailed POS is “numeral”, then apply the rules in Table 4.

**Table 4:** Rules used for our rule-based method, original Japanese with English translations

Option 1	main rule		Option 2
翌 (next)	一昨年	two years ago	より (from)
前 (before)	昨年	last year	まで (until)
入院前 (before hospitalization)	先月	last month	代 (‘s)
入院後 (after hospitalization)	先週	last week	前半 (early)
来院から (after visit)	昨日	yesterday	後半 (last)
午前 (a.m.)	今年	this year	-- (from)
午後 (p.m.)	今月	this month	-- (from)
発症から (after onset)	今週	this week	以上 (over)
発症してから (after onset)	今日	today	以下 (under)
治療してから (after care)	本日	today	から (from)
	来年	next year	時 (when)
	来月	next month	頃 (about)
	来週	next week	ごろ (about)
	翌日	tomorrow	ころ (about)
	再来週	the week after next	上旬 (early)
	明後日	day after tomorrow	中旬 (mid)
	同年	same year	下旬 (late)
	同月	same month	春 (spring)
	同日	same day	夏 (summer)
	翌年	following year	秋 (fall)
	翌日	the next day	冬 (winter)
	翌朝	the next morning	朝 (morning)
	前日	the previous day	昼 (noon)
	未明	early morning	夕 (evening)
	その後	after that	晩 (night)
	xx年	xx (year)	早朝 (early morning)
	xx月	xx (month)	明朝 (early morning)
	xx週間	xx (week)	以前 (before)
	xx日	xx (day)	以降 (after)
	xx時	xx (o’clock)	夕刻 (evening)
	xx分	xx (minutes)	ほど (about)

#### Hospital

If one of following keywords appeared in x, then mark it as *hospital*: 近医 (a near clinic or hospital), 当院 (this clinic or hospital), or 同院 (same clinic or hospital).

If x’s POS is “noun” and if detailed POS is not “non-autonomous word”, or if x is either “●”, “◯”, “▲” or “■” (these symbols are used for manual de-identification because the datasets are dummy EHRs), and if suffix of x is one of the following keywords, then mark it as *hospital*: 病院 (hospital or clinic), クリニック (clinic), or 医院 (clinic).

#### Sex

If x is either 男性 (man), 女性 (woman), men, women, man, woman (in English), then mark it as *sex*.

#### Time

If x’s detailed POS is “numeral” and if x consists of four-digit-numbers+slash+two-or-one-digit-numbers (corresponds to “yyyy/mm”) or two-or-one-digit-numbers+slash+two-or-one-digit-numbers (corresponds to “mm/dd”), then mark it as *time*.

If x’s detailed POS is “numeral” and followed by either of 歳 (old), 才 (old), or代 (‘s), then mark it as *time*.

If x is followed further by either of “より”, “まで”, “前半”, “後半”, “以上”, “以下”, “時”, “頃”, “ごろ”, “ころ”, “から”, “前半から”, “後半から”, “頃から”, “ごろから”, or “ころから”, then include these words in the span of the marked *time* tag.

### CRF-based method

We implemented a CRF-based system because many participants used CRFs in the MedNLP-1 de-identification task, including the second-best team and the baseline system. The best participant used a rule-based system, as described previously. We used the MALLET^2^ library for CRF implementation. We defined five training features for each token^3^: part-of-speech (POS), detailed POS, character type (Hiragana, Katakana, Kanji, or Number), a binary feature whether a token is included in our user dictionary or not, and another binary feature whether a token is beginning of its sentence or not.

### LSTM-based method

**Fig. 1:**
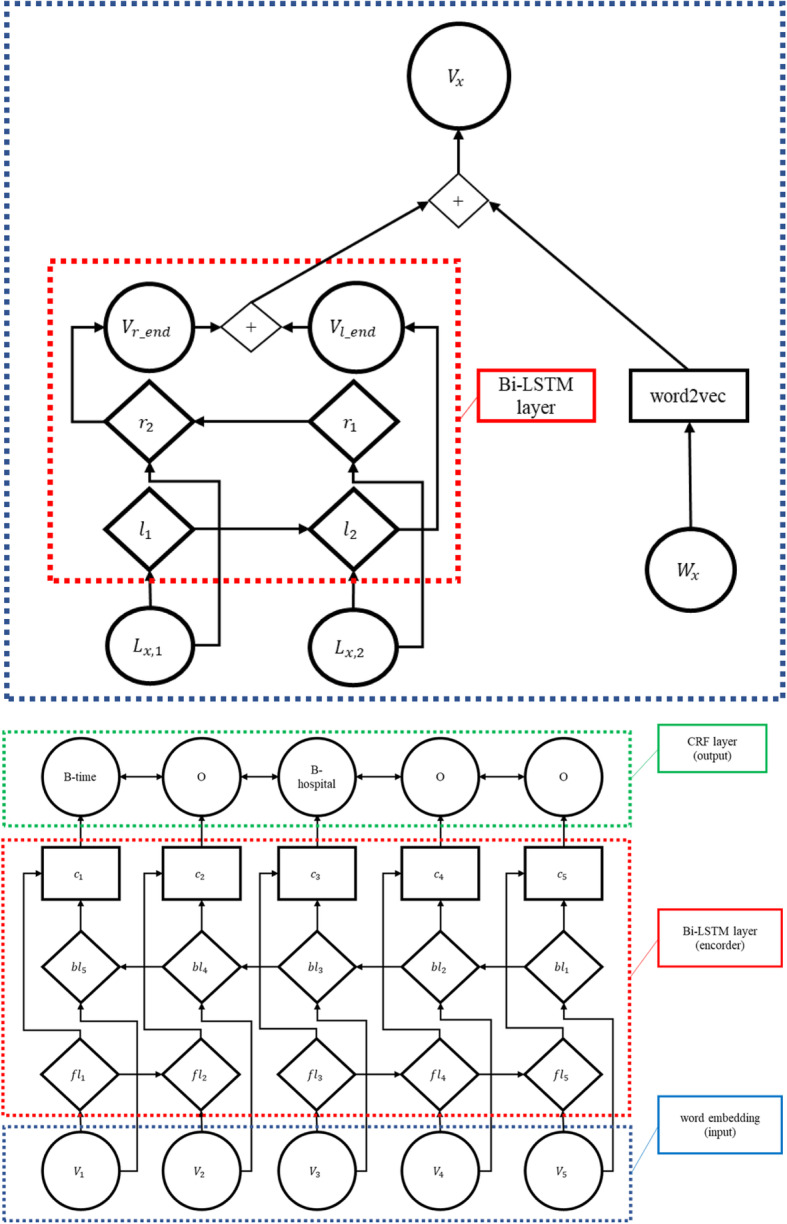
Conceptual figure of our LSTM-based model, showing embedding and NER in separate figures. + means concatenation. The first figure shows the embedding part, where *W*_*x*_ is an *x*^th^ input word, *L*_*x,i*_ is an *i*^th^ letter of the word *W*_*x*_, *r* denotes right to left (forward) LSTM, *l* denotes left to right (backward) LSTM, *V*_*x*_ is an intermediate node which corresponds to *W*_*x*_. The second figure shows the NER part, where *fl* denotes forward LSTM, *bl* denotes backward LSTM, *c* denotes concatenated vector, finally a CRF layer is shown with an example predicted named entities in the BIO annotation style

Our LSTM-based method combines bidirectional LSTM (bi-LSTM) and CRF, using character-based and word-based embeddings (Fig. 1) following earlier work that had been reported as successful for other languages [16].

For word-based embedding, we used the existing Word2Vec [23] model, which was trained using Japanese Wikipedia.^4^ We used bi-LSTM to embed characters; then we concatenated these two embeddings. This concatenated output was fed to another bi-LSTM and then sent to a CRF to output IOB tags.

Our implementation has been made publicly available in GitHub.^5^ Table 5 presents the parameter settings.

**Table 5:** LSTM parameter settings

Word embedding size	200
Character embedding size	100
Hidden layer of character	100
Hidden layer of LSTM	300
Learning rate	0.001

## Results

### Experiment settings and evaluation metrics

We followed the evaluation metrics of the MedNLP-1 shared task using IOB2 tagging [24]. We used four-fold cross validation, whereas the rule-based method requires no training data. We prepared five datasets: MedNLP (*MedNLP*), dummy EHRs (*dummy*), pathology reports (*pathology*), and MedNLP + dummy EHRs (*MedNLP + dummy*). We also prepared a dataset that comprises these three datasets (*all*). For each dataset, we applied cross validation. The CRF and LSTM are trained with three patterns of training data: the target dataset only, one of other datasets only, *MedNLP + dummy*, and *all*. Our evaluation uses a strict match of named entity spans, calculating F1-scores, precisions, and recalls. Table 6 presents the evaluation results.

**Table 6:** Evaluation results for each tag and in total, for different methods (rule, CRF, LSTM) and different evaluation datasets (MedNLP, dummy EHR, and pathology reports). *M*, *d*, and *P* respectively denote training data of MedNLP, dummy EHR, and Pathology reports; *M* + *d* denotes that training data consist of MedNLP+dummy EHR, *all* stands for all of these three datasets; other machine learning methods use the target evaluation dataset as its training data. In each cell, F1-score, precision, and recall are shown (in values multiplied by 100). The best scores for each tag type for each evaluation metric are presented in bold typeface. All evaluations were done by four-fold cross validations

Evaluation Results on MedNLP dataset													
tag type	#of tags	scores	Rule	CRF	CRF *d*	CRF *P*	CRF *M + d*	CRF *all*	LSTM	LSTM *d*	LSTM *P*	LSTM *M + d*	LSTM *all*
total	490	F1	**84.23**	82.62	43.85	0.71	26.40	67.34	83.07	41.26	0.43	67.35	57.03
		prec	78.90	**85.63**	46.20	2.50	21.51	66.54	81.33	41.07	0.48	66.98	57.94
		recall	**90.42**	79.95	42.33	0.41	59.76	68.38	86.12	41.57	0.38	68.17	56.34
age	56	F1	**93.43**	71.12	30.00	0.00	32.55	53.04	95.83	71.11	0.00	84.72	87.50
		prec	**96.00**	78.24	37.50	0.00	26.93	56.85	95.83	71.11	0.00	84.72	87.50
		recall	**91.16**	65.47	28.13	0.00	46.05	50.00	95.83	71.11	0.00	84.72	87.50
hospital	75	F1	84.73	**87.09**	43.25	0.00	26.02	70.04	66.67	13.33	13.89	66.67	41.67
		prec	80.75	**93.52**	66.67	0.00	20.55	91.67	75.00	11.11	10.67	70.83	45.83
		recall	**89.90**	81.71	27.50	0.00	53.06	60.42	62.50	16.67	20.00	63.89	38.89
person	0		N/A	N/A	N/A	N/A	N/A	N/A	N/A	N/A	N/A	N/A	N/A
sex	4	F1	**50.00**	16.67	16.67	0.00	14.65	25.00	0.00	20.00	0.00	25.00	25.00
		prec	**50.00**	25.00	12.50	0.00	8.68	25.00	0.00	20.00	0.00	25.00	25.00
		recall	**50.00**	12.50	25.00	0.00	50.00	25.00	0.00	20.00	0.00	25.00	25.00
time	355	F1	50.00	16.67	47.43	0.98	14.65	70.57	**96.14**	67.22	42.98	89.78	82.67
		prec	50.00	25.00	45.16	2.50	8.68	65.46	**95.00**	66.26	39.46	88.68	81.53
		recall	50.00	12.50	50.19	0.61	50.00	76.50	**97.41**	68.30	47.94	91.00	82.67
Evaluation Results on Pathology Report dataset													
tag type	#of tags	scores	Rule	CRF	CRF *M*	CRF *d*	CRF *M + d*	CRF *all*	LSTM	LSTM *M*	LSTM *d*	LSTM *M + d*	LSTM *all*
all	71	F1	13.97	74.26	0.00	0.62	1.45	57.63	**81.67**	0.00	0.00	1.45	81.25
		prec	8.65	86.72	0.00	1.47	10.00	64.98	**86.88**	0.00	0.00	10.00	82.48
		recall	43.33	65.16	0.00	0.39	0.78	54.06	78.84	0.00	0.00	0.78	**80.15**
age	0		N/A	N/A	N/A	N/A	N/A	N/A	N/A	N/A	N/A	N/A	N/A
hospital	31	F1	31.19	0.00	0.00	0.00	0.00	0.00	25.00	0.00	13.33	0.00	**58.33**
		prec	26.47	0.00	0.00	0.00	0.00	0.00	25.00	0.00	13.33	0.00	**58.33**
		recall	41.28	0.00	0.00	0.00	0.000	0.00	25.00	0.00	13.33	0.00	**58.33**
person	224	F1	0.00	91.08	0.00	0.00	6.25	71.31	95.19	0.00	0.00	0.00	**95.83**
		prec	0.00	**95.83**	0.00	0.00	10.00	74.79	95.19	0.00	0.00	0.00	**95.83**
		recall	0.00	87.21	0.00	0.00	4.55	69.63	95.19	0.00	0.00	0.00	**95.83**
sex	0		N/A	N/A	N/A	N/A	N/A	N/A	N/A	N/A	N/A	N/A	N/A
time	40	F1	9.25	10.57	0.00	2.00	0.00	18.82	**25.00**	3.81	0.00	6.25	19.44
		prec	5.25	16.67	0.00	1.79	0.00	20.83	**25.00**	6.67	0.00	10.00	19.44
		recall	**43.09**	9.09	0.00	2.27	0.00	19.32	25.00	2.67	0.00	4.55	19.44
Evaluation Results on Dummy EHR dataset													
tag type	#of tags	scores	Rule	CRF	CRF *M*	CRF *P*	CRF *M + d*	CRF *all*	LSTM	LSTM *M*	LSTM *P*	LSTM *M + d*	LSTM *all*
total	3017	F1	43.74	66.97	44.01	19.67	67.13	65.79	63.99	20.33	1.60	**69.82**	68.19
		prec	42.89	66.77	67.35	56.72	67.60	68.27	68.76	26.68	2.22	72.79	**80.26**
		recall	44.75	**67.34**	33.28	12.34	66.69	63.63	60.20	17.03	1.25	67.24	60.04
age	39	F1	**51.13**	48.46	29.35	0.00	38.87	33.82	50.00	22.38	0.00	50.00	41.67
		prec	51.97	**65.25**	28.85	0.00	41.56	35.72	50.00	19.05	0.00	50.00	45.83
		recall	50.46	**53.74**	30.00	0.00	36.71	32.50	50.00	32.38	0.00	50.00	41.67
hospital	170	F1	15.98	47.85	33.19	0.00	**48.62**	35.73	22.22	35.79	0.00	40.00	43.33
		prec	10.07	**53.18**	38.75	0.00	44.91	35.90	28.33	34.48	0.00	37.50	45.83
		recall	39.06	43.73	29.42	0.00	53.60	37.81	29.17	37.33	0.00	**43.75**	41.67
person	135	F1	0.00	26.96	0.00	0.00	28.36	15.48	**50.00**	0.00	0.00	45.83	37.50
		prec	0.00	26.79	0.00	0.00	29.91	19.64	**50.00**	0.00	0.00	45.83	37.50
		recall	0.00	30.71	0.00	0.00	27.99	13.39	**50.00**	0.00	0.00	45.83	37.50
sex	16	F1	**93.75**	35.92	29.17	0.00	90.08	33.93	0.00	40.00	0.00	50.00	50.00
		prec	**100.0**	44.27	50.00	0.00	95.83	50.00	0.00	40.00	0.00	50.00	50.00
		recall	**90.00**	43.13	20.83	0.00	85.63	27.08	0.00	40.00	0.00	50.00	50.00
time	2657	F1	49.48	71.28	42.14	21.20	70.60	68.33	83.93	51.97	48.89	85.70	**88.20**
		prec	51.81	71.44	64.94	59.35	71.24	70.94	84.82	52.59	48.89	86.51	**89.24**
		recall	47.38	71.15	32.08	13.58	70.00	66.08	83.29	51.46	48.89	84.93	**87.23**

### Results obtained using the MedNLP dataset

In this MedNLP dataset, the total number of *sex* is very small; that of *person* is zero. The *rule*-based system performed best in terms of the F1-score because its rules were tuned originally to the very MedNLP dataset. *LSTM* performed best for *age* and *time*, probably because these tags exhibit typical patterns of less variation. *LSTM* is superior to *Rule*, except for *sex* and *hospital.* Regarding *sex,* we observe better performance when *LSTM* uses more training data. Therefore, the data size is expected to have been the reason why *LSTM* was not good in *sex*.

### Results obtained using the dummy EHR dataset

*LSTM (M + d)* performed best in terms of the F1-score. *CRF* performed better when trained by *M + d* dataset than with the target dataset only. This performance increase consists of decrease of *age* and increase of all other tags, suggesting that these two datasets differ in their *age* tag annotation scheme.

The overall performance of this dummy EHR dataset is worse than the MedNLP dataset, suggesting that the dummy EHR dataset is more difficult to de-identify.

### Results obtained using the pathology report dataset

The LSTM-based method was better (81.67) than the CRF-based method (74.26), as shown by the 7.41 point F1-score when applied to our Pathology Report dataset.

Our rule-based system achieved very high recall, but very low precision scores for *time*, exhibiting a difference by 38 points. The pathology reports include many clinical inspection values written in an “xx/yy” format, which might engender confusion with dates expressed in an “mm/dd” format. We applied a workaround to limit [1 < = mm < = 12] and [1 < = dd < = 31], but it was insufficient: we need contextual information, not just rules. In addition, *hospital* is better than *time*, with less difference (15 points) of precision and recall.

When trained with the Pathology Report dataset only, its performance is better than our rule-based system. When trained with the *M + d* dataset, which does not contain the pathology dataset, neither CRF nor LSTM works fine because the pathology reports differ greatly in terms of their styles of description and named entities.

## Discussion

These results suggest that our datasets have quite different characteristics in what context and in what form their named entities appear, but LSTM adapted to these differences well. Adding the Pathological Report dataset to the training data seems to degrade the system performance for other target test datasets because of the different dataset characteristics (examples presented in Table 1). For example, when trained with the Pathological Report dataset, the *hospital* tags of the MedNLP dataset show lower performance because of the different descriptions of hospital names among these two datasets. The Pathological Report dataset has full hospital names such as “Shizudai Dermatology Clinic,” but the other two datasets have more casual descriptions such as “近医 (hospital nearby)” and “当院 (our hospital)”. The Pathology Report dataset has different contextual patterns that could have learned by machine learning methods such as “院外標本 (ex-hospital sample)” immediately before hospital tags, and a suffix/prefix such as “xx hospital” or “xx clinic”. These words, “hospital” and “clinic”, might have been learned as semantically similar by Word2Vec.

Another difference of datasets is the coherence of annotations. We compared the original annotations of the datasets with our own new annotations created for this study by three annotators. These new annotations were created to calculate inter-annotator agreement as described in the Dataset section. The original versus new inter-annotator agreement (and inter-annotator agreement of the three annotators) in average F1-scores were 0.566 (0.861), 0.342 (0.761), and 0.772 (0.802), respectively, for the MedNLP, Dummy, and Pathology Report datasets. As these scores strongly suggest, the original annotations were insufficiently coherent. By contrast, our new annotations are much more coherent because we have included more detailed annotation guidelines. For example, our guidelines include specifications of prefixes, suffixes and classifiers.. Annotating larger datasets with this coherent guideline is anticipated as a subject for future work. It is particularly interesting that our system performance was better than the inter-annotator agreement in the Pathology Report dataset. One reason is expected to be the remaining vague part of the guideline, such as inclusion of particles when assigning named entities. We applied the automatic tagger for pre-annotation; then human annotators reviewed the results. However, annotators sometimes overly depend on automatically annotated parts-of-speech without considering the context and semantics; alternatively, the part-of-speech tagger can simply fail. Therefore, an annotation guideline including precise part-of-speech specifications will be required.

An earlier study that applied a similar LSTM-based method to de-identify English medical data [25] found lower F1-scores for LOCATION and NAME tags on the i2b2 2014 dataset and MIMIC-III dataset [26], which includes records of 61,532 patients in an intensive care unit (ICU); performance of naïve CRF was very low. This LOCATION tag corresponds to our *hospital* tag, exhibiting similar characteristics among different languages. The LSTM-based method can be regarded as effective in Japanese medical de-identification tasks as well. If a larger dataset were available, then it would yield better performance.

Japanese-specific issues include the following difficulties: Japanese (and Chinese) have no spaces between tokens, which makes tokenization much more difficult and ambiguous. The number of letter types is much greater than in other languages, including tens of thousands of kanji letters, 50 hiragana letters, 50 katakana letters, numerals, and alphabets. The languages also have more synonyms than in other languages.

Our system performance almost reaches to the inter-annotator agreement, which can be regarded as upper bound of system performance. The current performances are sufficiently high compared to other publicly available Japanese de-identification tools. Therefore, we plan to apply our system to actual de-identification tasks in hospitals.

## Conclusions

We implemented three de-identification methods for Japanese EHRs and applied these methods to three datasets, which are derived from two dummy EHR sources and one real Pathology Report dataset. These datasets have manually annotated de-identification tags, following the MedNLP shared task annotation guideline.

Our best F1-scores over all the tag types are 84.23 (rule-based), 68.19 (LSTM), and 81.67 (LSTM) points, respectively, for the MedNLP dataset, the dummy EHR dataset, and the Pathology Report dataset. Our LSTM-based method performed best in two datasets, whereas our rule-based method performed best in the MedNLP dataset. However, our LSTM-based method also achieved a good score of 83.07 points in the MedNLP dataset, which only differs 1.16 points from the best score of the rule-based method. Our results demonstrate that the bi-LSTM based method with character-embedding and word-embedding tends to work better than other methods, exhibiting more robustness than CRF over different data sources. The LSTM-based method was better than the CRF-based method, exhibiting a 7.41 point F1-score difference when applied to our Pathology Report dataset. This report is the first describing a study applying this LSTM-based method to any de-identification task of Japanese EHRs.

Machine learning methods can extract named entities of de-identification comparable to a rule-based method that is tuned manually to specific target data. However, machine learning methods are still less adequate for application to expressions with low occurrence. Probably because of the insufficient data size, our methods yielded worse evaluation scores than were obtained with the other languages when applied to the i2b2 task and MIMIC-III. Combinations of LSTM and rule-based methods are left as a subject for future work.

The current performance is sufficiently high among publicly available Japanese de-identification tools. Therefore, we plan to apply our system to actual de-identification tasks in hospitals. Although it is still difficult to make real EHRs publicly available, we could use our large amount of EHRs inside our hospitals. Increasing the size of annotated datasets for such internal usage is left as another subject for future work.

## Data Availability

The source code will be made available on the web; datasets will be made partially available.

## Abbreviations

NLP: natural language processing
LSTM: Long Term Short Memory: a kind of recurrent neural network
CRF: Conditional Random Field: a kind of machine learning method
POS: part-of-speech
EHR: electronic health record

## Footnotes

1 https://www.atilika.com/ja/kuromoji/

2 http://mallet.cs.umass.edu/

3 Hereinafter, “token” means a “morpheme” of the Japanese language, which does not have any space between tokens. A “morpheme” is the smallest meaningful unit in a language.

4 http://www.cl.ecei.tohoku.ac.jp/~m~suzuki/jawiki_vector/

5 https://github.com/johokugsk

## References

[1] Act on the Protection of Personal Information. Japan, 2003..

[2] Mullner R, Rafalski EM (1996). Health insurance portability and accountability act of 1996 (HIPAA).

[3] Act on Anonymously Processed Medical Information to Contribute to Medical Research and Development. Japan, 2017.

[4] Stubbs A, Kotfila C, Uzuner Ö (2015). Automated systems for the de-identification of longitudinal clinical narratives: overview of 2014 i2b2/UTHealth shared task track 1. J Biomed Inform.

[5] Grouin C, Zweigenbaum P (2013). Automatic De-identification of French clinical records: comparison of rule-based and machine-learning approaches. Stud Health Technol Inform.

[6] Grouin C, Névéol A (2014). De-identification of clinical notes in French: towards a protocol for reference corpus development. J Biomed Inform.

[7] Dalianis H, Hassel M, Velupillai S (2009). The Stockholm EPR corpus – Characteristics and some initial findings. Proceedings of the 14th International Symposium Health Informatics Management Research.

[8] Dalianis H, Velupillai S (2010). De-identifying Swedish clinical text – refinement of a gold standard and experiments with conditional random fields. J Biomed Sem.

[9] Jian Z, Guo X, Liu S, Ma H, Zhang S, Zhang R, Lei J (2017). A cascaded approach for Chinese clinical text de-identification with less annotation effort. J Biomed Inform.

[10] Du L, Xia C, Deng Z, Lu G, Xia S, Ma J (2018). A machine learning based approach to identify protected health information in Chinese clinical text. Int J Med Inform.

[11] Morita M, Kano Y, Ohkuma T, Miyabe M, Aramaki E (2013). Overview of the NTCIR-10 MedNLP Task. Proceedings of the NTCIR-10 conference.

[12] Aramaki E, Morita M, Kano Y, Ohkuma T (2014). Overview of the NTCIR-11 MedNLP-2 Task. Proceedings of the NTCIR-11 conference.

[13] Cortes C, Vapnik V (1995). Support-vector networks. Mach Learn.

[14] Lafferty J, McCallum A, Pereira F (2001). Conditional random fields : Probabilistic models for segmenting and labeling sequence data. Proceedings of the Eighteenth International Conference on Machine Learning (ICML 2001).

[15] Hochreiter S, Schmidhunber J (1997). Long short-term memory. Neural Comput.

[16] Lample G, Ballesteros M, Subramanian S, Kawakami K, Dyer C (2016). Neural Architectures for Named Entity Recognition. Proceedings of the 15th Annual Conference of the North American Chapter of the Association for Computational Linguistics: Human Language Technologies (NAACL-HLT 2016).

[17] Sang E (2002). Introduction to the CoNLL-2002 Shared Task: Language-independent Named Entity Recognition. Proceedings of the Sixth Conference on Natural Language Learning (CoNLL 2002).

[18] Sang E, Fen M, Hovy E (2016). Introduction to the CoNLL-2003 Shared Task: Language-Independent Named Entity Recognition. Proceedings of the Seventh Conference on Natural Language Learning (HLT-NAACL 2003).

[19] Misawa S, Taniguchi M, Miura Y, Ohkuma T (2017). Character-based Bidirectional LSTM-CRF with words and characters for Japanese Named Entity Recognition. Proceedings of the First Workshop on Subword and Character Level Models in NLP (SCLeM 2017), 2017 Conference on Empirical Methods in Natural Language Processing (EMNLP 2017).

[20] Kajiyama K, Horiguchi H, Okumura T, Morita M, Kano Y. De-identifying Free Text of Japanese Dummy Electronic Health Records. In: Proceedings of the Ninth International Workshop on Health Text Mining and Information Analysis (LOUHI 2018), 2018 Conference on Empirical Methods in Natural Language Processing (EMNLP 2018). 2018. p. 65–70.

[21] Hatano K, Ohe K (2003). Information retrieval system for Japanese Standard Disease-code Master Using XML Web Service. Proceedings of the American Medical Informatics Association (AMIA) Annual Symposium.

[22] Imaichi O, Yanase T, Niwa Y (2013). A Comparison of Rule-Based and Machine Learning Methods for Medical Information Extraction. Proceedings of the First Workshop on Natural Language Processing for Medical and Healthcare Fields, The Sixth International Joint Conference on Natural Language Processing (IJCNLP 2013).

[23] Mikolov T, Sutskever I, Chen K, Corrado G, Dean J (2013). Distributed Representations of Words and Phrases and their Compositionality. Proceedings of the Advances in Neural Information Processing Systems 26 (NIPS 2013).

[24] Sang E, Veenstra J (1999). Representing text chunks. Proceedings of the Ninth Conference of the European Chapter of the Association for Computational Linguistics (EACL 1999).

[25] Dernoncourt F, Lee JY, Uzuner O, Szolovits P (2017). De-identification of patient notes with recurrent neural networks. J Amer Med Info Assoc.

[26] Johnson A, Pollard T, Shen L, Lehman L, Feng M, Ghassemi M, Moody B, Szolovits P, Celi L, Mark R (2016). MIMIC-III, a freely accessible critical care database. Sci Data.

